# Chemical Composition and Antiproliferative Effects of a Methanol Extract of* Aspongopus chinensis* Dallas

**DOI:** 10.1155/2019/2607086

**Published:** 2019-05-30

**Authors:** Jun Tan, Ying Tian, Renlian Cai, Rui Luo, Jianjun Guo

**Affiliations:** ^1^Provincial Key Laboratory for Agricultural Pest Management of Mountainous Region, Institute of Entomology, Guizhou University, Guiyang, Guizhou 550025, China; ^2^Department of Histology and Embryology, Zunyi Medical University, Zunyi, Guizhou 563000, China; ^3^College of Life Science, Guizhou University, Guiyang, Guizhou 550025, China

## Abstract

Natural products from insects can be potent sources for developing a variety of pharmaceutical products.* Aspongopus chinensis* Dallas has been used as a traditional Chinese medicine and there are several clinical evidences to support its anticancer activity. However, the anticancer active ingredients present in* A. chinensis* remain unidentified. In the present study, we investigated the anticancer effects of a methanol extract of* A. chinensis *(AME). Gas chromatography mass spectrometry was used to analyse the chemical composition of AME. The cell viability of MDA-MB-453 and HCC-1937 cells treated with different concentrations of AME was detected by MTT assay and the ratio of cells in different cell cycle phases was analysed by flow cytometry. The expression of genes associated with cell cycle was analysed by real-time PCR assay. The results showed that oleic acid (25.39%) and palmitic acid (21.798%) are the main anticancer compounds present in AME. There was a concentration-dependent decrease in the proliferation of MDA-MB-453 and HCC-1937 cells. Moreover, treatment with AME induced a S-phase arrest in the cells. Real-time PCR assay demonstrated that AME could significantly downregulate the expression of* CDC20*,* AURKB*,* PLK1*,* CCNB2*, and* TOP2A* mRNAs and upregulate the expression of* GADD45A* mRNA. We demonstrate that the methanol extract of* A. chinensis *could be a potential natural alternative or complementary therapy for breast cancer.

## 1. Introduction

Breast cancer is the most common malignancy in women and accounts for around 18% of all cancers in females [1]. In the United States, about 63,960 cases of female breast carcinoma were diagnosed in 2018. In women, the three most common types of cancers are breast, lung, and colorectal cancer. Breast cancer alone accounts for 30% of new cancer diagnoses in women [2]. Such cases are particularly on the rise in some Asian countries. In the past decade, the incidence of breast cancer in China has increased by 27%, and 40% of the diagnosed patients died within 5 years. The incidence of breast cancer in Chinese women and the mortality associated with it has increased sharply [3]. The treatment of cancer with traditional Chinese medicine (TCM) has attracted extensive attention, as chemotherapeutic agents have more side effects [4].

*Aspongopus chinensis *Dallas (Dinidoridae, Insecta) is a TCM [5]. Owing to its anticancer, antibacterial, and anticoagulant functions, much attention has been focused on* A. chinensis *[6]. It can be used to treat gastric and oesophageal cancers. Trichloromethane extracts and water extracts from* A. chinensis* could inhibit the proliferation of human gastric (SGC-7901) and liver (HepG2) cancer cells [7–10]. Serum containing* A. chinensis* could inhibit the proliferation of SW480 [11]. Our previous findings revealed that the haemolymph from* A. chinensis* had a significant inhibitory effect on the proliferation of SGC-7901, BGC-823, and MCF-7 cells [12–15] and that the apoptosis of SGC-7901 and MCF-7 cells induced by the haemolymph might be through mitochondrial signalling pathway [16]. Excellent anticancer effects of* A. chinensis* have been supported by an increasing body of evidence; however, the effective ingredients present in it have not been identified as yet.

In the current study, the chemical composition of an* A. chinensis* methanol extract (AME) and its anticancer activities were investigated. The results of this study support the opinion that* A. chinensis* is a valuable insect resource that can be investigated further for potential medicinal uses.

## 2. Materials and Methods

### 2.1. Chemicals and Reagents

The 3-(4,5-dimethylthiazol-2-yl)-2,5-diphenyltetrazolium bromide (MTT) and cell cycle detection kit were obtained from Beyotime (Beyotime Institute of Biotechnology, Shanghai, China). L-15 medium, RPMI-1640 medium, and fetal bovine serum (FBS) were purchased from Hyclone (GE Healthcare, Logan, UK). Dimethyl sulfoxide (DMSO) was obtained from Sigma company.

### 2.2. Materials

Specimens of* A. chinensis* (known by its name in Chinese as Jiuxiangchong) were collected in Zunyi, Guizhou Province, China (27°42′15′′ N latitude, 106°55′22′′ E longitude, 1161 m a.s.l. altitude). The specimens were authenticated by Prof. Zizhong Li (Institute of Entomology, Guizhou University, Guiyang, China). They were shade dried and ground into a fine powder, which was then extracted with 5 × the volume of methanol for 48 h at 25°C. The insoluble particles were removed by centrifugation at 12,000 ×* g* for 10 min at 4°C and by filtration through a 0.22-*μ*m membrane (Millipore, Merck Millipore Ltd., Germany). The bulk methanol extracts were vacuum dried in a freeze dryer (Chaist, Alpha 1-2 LD plus, Germany) and quantified. For* in vitro* cellular assay, DMSO was used to redissolve the dried methanol extracts, which were stored at −80°C until use. The final concentration of DMSO in the cell culture studies was maintained at 0.1%.

### 2.3. Cell Culture

Human breast cancer cell lines MDA-MB-453 and HCC-1937 were kindly provided by the Stem Cell Bank, Chinese Academy of Sciences. The cells were cultured in L-15 and RPMI-1640 media (GE Healthcare, Logan, UK), supplemented with 2 mM L-glutamine and 10% FBS (Life Technology, Shanghai, China), containing penicillin (100 U/mL) and streptomycin (100 *μ*g/mL, Life Technology, Shanghai, China). The HCC-1937 cells were cultured in an incubator with 5% CO_2_ at 37°C and MDA-MB-453 cells were cultured in an incubator at 37°C without CO_2_ (air, 100%).

### 2.4. Cell Viability Assay

The MDA-MB-453 and HCC-1937 cells (5 × 10^3^/well) were seeded into 96-well plates and incubated for 24 h. After treatment with AME (0.5, 1, or 1.5 mg/mL) or an equivalent concentration (1%) of DMSO for 48 h, the cell viability was determined by MTT assay. MTT solution (20 *μ*L, 5 mg/mL) was added to the wells, and the cells were incubated for 4 h. Thereafter, the MTT solution was removed and DMSO (150 *μ*L) was added to each well. The absorbance was measured at 570 nm using an automated microplate reader (Bio-Rad, Hercules, CA, USA). The control values for DMSO were deducted from the absorbance values obtained for all the groups. The 50% growth inhibition value (IC50) of AME was calculated. The experiment was repeated five times. The cell survival rate was calculated on the basis of the percentage of cell survival in the drug-treated group versus that in the control group. The formula used for calculation was as follows: cell viability (%) = absorbance (treated)/absorbance (control) × 100.

### 2.5. Morphological Study

The MDA-MB-453 and HCC-1937 cells were grown on coverslips (1 × 10^5^ cells/cover slip) and incubated with different concentrations (0.5, 1, or 1.5 mg/mL) of AME or with 0.1% DMSO for 48 h, and then fixed in a solution of ethanol: acetic acid (3:1, v/v). The cover slips were mounted on glass slides for morphometric analysis. Three monolayers per experimental group were photographed. Morphological changes in MDA-MB-453 and HCC-1937 cells were determined using a Nikon bright-field inverted light microscope (Tokyo, Japan) at 200× (for MDA-MB-453 cells) or 400× (for HCC-1937 cells) magnification.

### 2.6. Cell Cycle Assay

Cell cycle analysis was performed using a cell cycle detection kit (Beyotime Biotechnology, Shanghai, China), according to the manufacturer's instructions. Briefly, the MDA-MB-453 and HCC-1937 cells were seeded in 6-well plates (1 × 10^5^/well) and cultured for 24 h. The cells were starved by culturing them in serum-free medium for 12 h. After treatment with AME (0 and 2.57 mg/mL for MDA-MB-453 cells; 0 and 3.64 mg/mL for HCC-1937 cells), the cells were further cultured for 48 h. Thereafter, the cells were collected by trypsinization with EDTA-free trypsin, washed with ice-cold PBS three times, and fixed in 70% ice-cold ethanol for 12 h at 4°C. Subsequently, the cells were washed thrice with PBS and stained using a solution containing PI and RNase A for 30 min in the dark. The cell cycle distribution was determined using a flow cytometer (Beckman Coulter Epics XL, USA). Each experiment was performed in triplicate.

### 2.7. RNA Extraction and Real-Time RCR Analysis

After AME treatment, differences in the expression of genes in MDA-MB-453 were detected by real-time PCR assay. Total RNA was extracted from MDA-MB-453 cells after 48 h of treatment with AME (0 and 2.57 mg/mL) using TRIzol reagent (Ambion), according to the manufacturer's protocol. The cDNA synthesis reaction was done using 0.5 *μ*g total RNA for each sample, using the cDNA SuperScript First-Strand Synthesis kit (Takara Biotechnology Co., Ltd.). Real-time PCR was performed using the SYBR Green PCR kit (Takara Biotechnology Co., Ltd.). qPCR mixture included the following: 10.0 *μ*L SYBR Green Mix, 10 *μ*L of 5 M forward and reverse primers, 1.0 *μ*L template cDNA, and 8 *μ*L ddH_2_O. The reaction conditions were as follows: 95°C for 30 s, 40 cycles of denaturation at 95°C for 5 s, annealing at 65°C for 30 s, and extension at 72°C for 35 s. The sequences of the primers for the tested genes are presented in Table 1. GAPDH was used as the reference gene and the threshold cycle number (CT) was recorded for each reaction.

### 2.8. Gas Chromatography Mass Spectroscopy (GC-MS)

GC-MS analysis was performed to determine the molecular composition of AME. The filtered AME was analysed using Agilent 6890 Gas Chromatograph with a 5975C Mass-selective Detector (Agilent, Palo Alto, CA, USA). The column used was ZB-5 MSI (30 m × 0.25 mm × 0.25 *μ*m) and the mobile phase was 5% phenyl/95% dimethylpolysiloxane. Helium was used as the carrier gas at a constant flow rate of 1 mL/min. The initial oven temperature was 40°C and it was maintained at this temperature for 1.5 min; the temperature was gradually increased to 230°C at a rate of 4°C/min and was maintained for 8 min. The temperature of the injection port was 250°C and the flow rate of helium was 1 mL/min. The compounds discharged from the column were detected by a quadrupole mass detector. The ions were generated by electron ionization method. The temperatures of the MS quadrupole and source were 150 and 230°C, respectively, electron energy was 70 eV, temperature of the detector was 230°C, the emission current multiplier voltage was 1624 V, the interface temperature was 280°C, and the mass range was from 20 to 460 u. The relative mass fraction of each chemical component was determined by peak area normalization method. The National Institute Standard and Technology Library was used to analyse the spectrum and identify the compounds detected.

### 2.9. Data Analysis

All results were expressed as means ± SD. SPSS 18.0 software (IBM, Chicago, IL, USA) was used for one-way ANOVA to evaluate the significance of the differences between groups. A value of* P* < 0.05 was defined as significant.

## 3. Results

### 3.1. AME Inhibits the In Vitro Growth of MDA-MB-453 and HCC-1937 Cells

**Figure 1:**
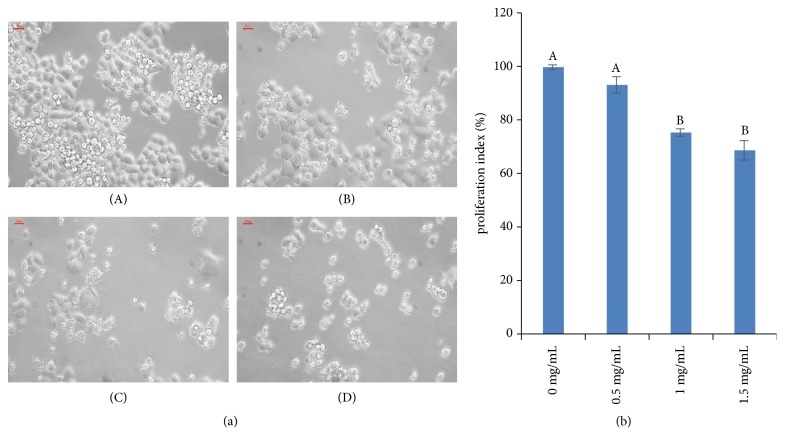
Effect of* Aspongopus chinensis *methanol extract (AME) on the proliferation of breast cancer MDA-MB-453 cells. (a) Morphologic characterization of MDA-MB-453 cells treated with different concentrations of AME (0, 0.5, 1, or 1.5 mg/mL (A-D), respectively; 200×). (b) Viability of MDA-MB-453 cells treated with 0 (control), 0.5, 1, or 1.5 mg/mL AME. Values are presented as the mean ± SD of five independent experiments. Different upper case letters indicate significant difference between groups (Fisher's LSD,* P*<0.01).

**Figure 2:**
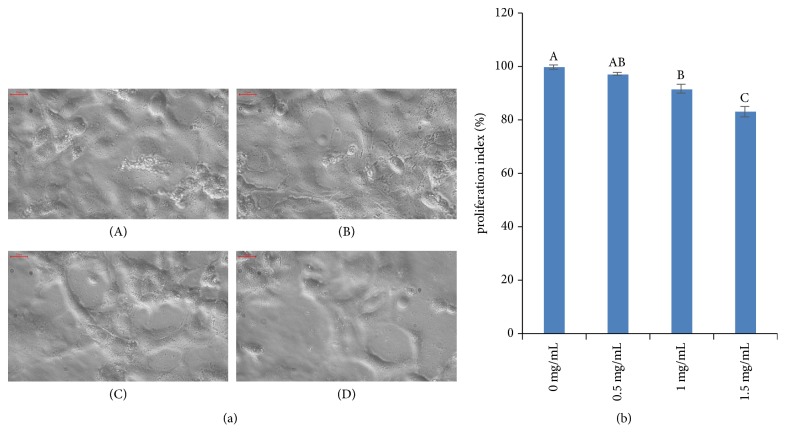
Effect of* Aspongopus chinensis *methanol extract (AME) on the proliferation of breast cancer HCC-1937 cells. (a) Morphologic characterization of HCC-1937 cells treated with different concentrations of AME (0, 0.5, 1, or 1.5 mg/mL (A-D), respectively; 400×). (b) Viability of HCC-1937 cells treated with 0 (control), 0.5, 1, or 1.5 mg/mL AME. Values are presented as the mean ± SD of five independent experiments. Different upper case letters indicate significant difference between groups (Fisher's LSD,* P*<0.01).

Morphological characterization of MDA-MB-453 and HCC-1937 cells was performed after treatment with AME. The extent of cell shrinkage at 48 h of treatment with different concentrations of the AME was evaluated by light microscopy. The results were compared with those obtained for untreated breast cancer cells. The MDA-MB-453 cells appeared to be more susceptible to AME; the most recognizable morphological changes in AME-treated MDA-MB-453 cells were cytoplasmic condensation and shrinkage of cell membrane (Figure 1(a)). In contrast, the HCC-1937 cells showed no distinct morphological changes upon treatment and only a decrease in the number of cells was observed (Figure 2(a)).

The results of viability assay for MDA-MB-453 and HCC-1937 cells treated with different concentrations (0.5, 1, or 1.5 mg/mL) of AME or DMSO (0.1%) for 48 h are shown in Figures 1(b) and 2(b). The viability of MDA-MB-453 cells at 0.5, 1, and 1.5 mg/mL doses was 96.39% ± 3.06%, 75.42% ± 1.40%, and 68.50% ± 3.65%, respectively (Figure 1(b)). The viability of HCC-1937 cells at 0.5, 1, and 1.5 mg/mL doses was 97.14% ± 0.65%, 91.61% ± 1.58%, and 82.96 ± 1.98%, respectively (Figure 2(b)). A significant reduction in the survival of both the cell lines was observed at 1 and 1.5 mg/mL doses. The results revealed that AME reduced the survival of both the cells in a concentration-dependent manner (*P* < 0.01), although MDA-MB-453 (IC50 = 2.57 mg/mL) was more sensitive than HCC-1937 cells (IC50 = 3.64 mg/mL).

### 3.2. AME Induces S-Phase Arrest of MDA-MB-453 and HCC-1937 Cells

**Figure 3:**
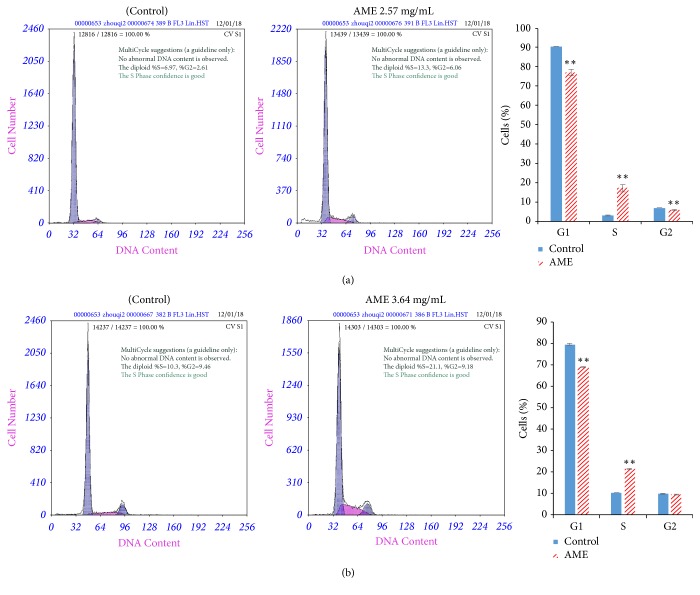
Cell cycle arrest in the S-phase in MDA-MB-453 and HCC-1937 cells treated with* Aspongopus chinensis *methanol extract (AME). (a) MDA-MB-453 cells were treated with 2.57 mg/mL AME for 48 h; (b) HCC-1937 cells were treated with 3.64 mg/mL AME for 48 h. Data are representative of three independent experiments.  ^*∗∗*^*P* < 0.01.

To investigate the factors contributing to the growth inhibition of MDA-MB-453 and HCC-1937 cells, we studied the effect of AME on cell cycle distribution in both the cell lines. We found that treatments of both the cell lines with AME resulted in cell cycle arrest in the S-phase (Figure 3). For MDA-MB-453 cells, the percentage of cells in the S-phase increased from 2.83% for untreated cells to 17.13% for cells treated with 2.57 mg/mL AME (Figure 3(a)). For HCC-1937 cells, the percentage of cells in the S-phase increased from 10.43% to 21.40% for cells treated with 3.64 mg/mL AME (Figure 3(b)). The increase in the percentage of cells in the S-phase also caused a significant reduction in the percentage of cells in the G1-phase (Figure 3).

### 3.3. AME Downregulates the Expression of CDC20, AURKB, PLK1, CCNB2, and TOP2A mRNAs

**Figure 4:**
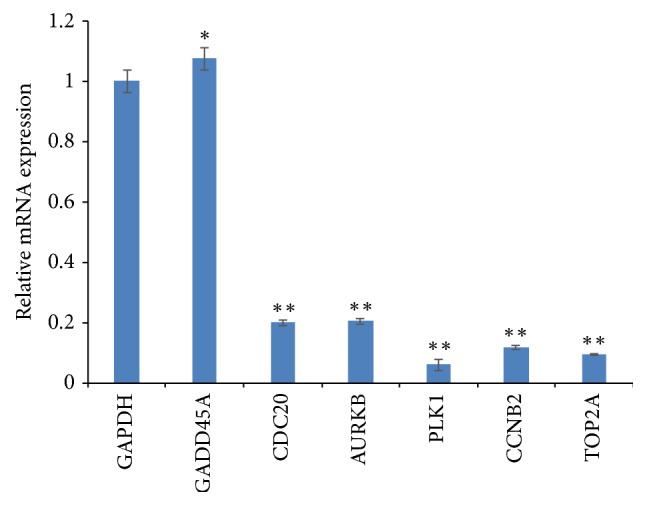
Relative mRNA expression of target genes in MDA-MB-453 cells treated with AME at 2.57 mg/mL for 48 h. Data are representative of three independent experiments.  ^*∗∗*^*P* < 0.01;  ^*∗*^*P* < 0.05.

The relative expression levels of target genes modulated by AME were determined by qPCR assay. As shown in Figure 4, AME could significantly downregulate the expression of* CDC20*,* AURKB*,* PLK1*,* CCNB2*, and* TOP2A* mRNAs (*P* < 0.01) and upregulated the expression of* GADD45A* mRNA (*P *< 0.05) in MDA-MB-453 cells compared to the expression levels of the respective genes in the untreated group (Figure 4).

### 3.4. GC-MS Analysis of A. chinensis Extracts

**Figure 5:**
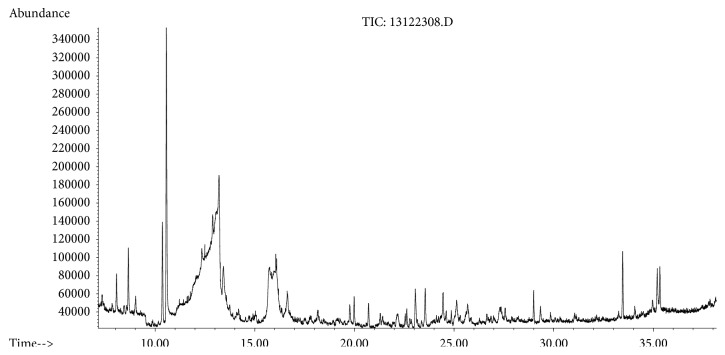
GC-MS chromatogram of compounds presents in the methanol extract of* Aspongopus chinensis *Dallas.

The GC-MS analysis was performed to determine the presence of potential anticancer or medicinally important compounds in the* A. chinensis *extract. A total of 16 compounds were identified (Figure 5), among which seven were esters, based on their peak area, retention time, and molecular formula. These esters were of oleic acid (25.39%), palmitic acid (21.798%), z-11-hexadecenoic acid (8.08%), threitol (6.74%), stearic acid (3.04%), 2-hexenoic acid (2.49%), and 1-(14-methylhexadecanoyl) pyrrolidine (0.34%) (Table 2); the percentage content of fatty acids in AME was 61.337%. The oleic acid and palmitic acid were determined to be the major compounds present in the* A. chinensis* extracts, and these have medically important functions (Table 2). Oleic acid is a fatty acid found in animal and vegetable oils; it is the major component of olive oil responsible for a healthy Mediterranean diet, and is especially effective for the prevention of breast cancer [17]. Palmitic acid, one of the most common saturated fatty acids in animals and plants, has been reported to induce apoptosis in exocrine pancreatic AR42J cells [18].

## 4. Discussion

Breast cancer is a major public health problem worldwide [1]. The traditional methods for breast cancer treatment mainly include surgery, chemotherapy, radiotherapy, and drug therapy. Among these, surgery is the preferred method. However, because of its high recurrence and metastasis rate, surgical resection of solid tumours is unable to achieve a radical effect. Chemotherapy and radiotherapy can cause vomiting, baldness, thrombocytopenia, and other adverse reactions. Therefore, searching for noncytotoxic anticancer drugs is an important area of research [4].* A. chinensis* has been used as a TCM and has been shown to have good clinical anticancer effects in China. Previous investigations have shown that* A. chinensis* possess anticancer, anticoagulant, and antibacterial effects [6]. However, the anticancer active ingredients present in it have remained undiscovered. 

In recent years, a lot of attention has been given to the isolation of natural products from* A. chinensis *[5, 19–21]. Four kinds of small molecules extracted from* A. chinensis* (a new oxazole and three known N-acetyldopamines) have been identified and were proven to inhibit the proliferation of cancer cells* in vitro *[22]. In a previous study, we determined that the content of proteins and fatty acids in* A. chinensis *were 23.68% and 27.72%, respectively, and unsaturated fatty acids occupied about 64.03% of the total fatty acid content. Our studies further showed that the proteins extracted from* A. chinensis* could inhibit the proliferation of SGC-7901 and MCF-7 cells [5, 13–16]. However, there is not much information on the effects of fatty acids present in* A. chinensis*.

Fatty acids are components of cell membranes. Altering the components of fatty acids in the membrane can inhibit DNA synthesis in cancer cells [23]. In the present study, oleic acid and palmitic acid were found to be the main compounds in the AME (Table 2). In recent years, palmitic acid has attracted wide attention because of its therapeutic roles in some chronic diseases, such as metabolic syndrome, diabetes, and inflammation [24]. Byberg* et al*. analysed the ratio of fatty acids in the blood of Swedish cancer population; the results showed that the imbalance of palmitic and palmitoleic acid metabolism would result in the death of cancer cells [25]. Unsaturated fatty acids have inhibitory effects on the occurrence, growth, metastasis, and proliferation of tumour cells [26, 27]. In the present study, we determined that the percentage content of fatty acids in AME was 61.337%. Moreover, we observed that AME could inhibit the proliferation of MDA-MB-453 and HCC-1937 cells (Figures 1 and 2). The proliferation of treated cells was reduced in a dose-dependent manner (*P* < 0.01); however, MDA-MB-453 (IC50 = 2.57 mg/mL) cells were more sensitive than HCC-1937 (IC50 = 3.64 mg/mL) cells. The MDA-MB-453 cells are HER-2 positive, and the overexpression of HER2/neu is associated with tumour classification and poor prognosis [28]. Moreover, oleic acid is reported to inhibit the HER-2/neu overexpression and promote apoptosis [17].

The regulation of cell cycle is very important for cell growth [29]. In the present study, we found that treatment of MDA-MB-453 and HCC-1937 cells with AME resulted in cell cycle arrest in the S-phase (Figure 3). Similar results have been reported for Berberine, Tanshinone I, and the ethanol extracts of* Ganoderma lucidum, *which could inhibit the proliferation of breast cancer MCF-7 cells and induce cell cycle arrest [30–32]. Elm Indican II and Berberine could inhibit the proliferation of breast cancer cells and induce cell cycle arrest in the G0/G1 and S-phase [33, 34]. Based on the results of these studies and our findings in the present study, we speculate that TCM may contain components that can induce cell cycle arrest in breast cancer cells. In addition, the expression levels of* CDC20, AURKB, PLK1, CCNB2*, and* TOP2A *were found to be downregulated by AME (Figure 4). CDC20 is a cell cycle protein and its overexpression can promote the proliferation and inhibit the apoptosis of cancer cells [35]. PLK1 is highly expressed in tumour cells [36] and is involved in almost all the processes of mitosis [37]. PLK1 can inhibit the activity of anaphase promoting complex by phosphorylating CDC20, which can promote cytokinesis. The inhibition of the activity of PLK1 can prevent cells from completing mitosis and eventually leads to cell death [38]. AURK family proteins are highly expressed in tumour cells, and their inhibition can decrease the proliferation of tumour cells [39]. CCNB2 is a cell cycle protein, which is highly expressed in cancer tissues [40]. TOP2A is a target of anticancer drugs; its downregulation can inhibit chromosome aggregation and assembly [41]. In the present study, we found that AME can cause S-phase arrest of breast cancer cells, downregulate the expression of* CDC20*,* AURKB*,* PLK1*,* CCNB2*, and* TOP2A*, and upregulate the expression of* GADD45A*, eventually inhibiting the proliferation of breast cancer cells. Although* A. chinensis *has been used as a TCM for hundreds of years in China and the long-term clinical application suggests its anticancer effects [8, 10], several questions about the ingredients with anticancer properties remain unanswered. The present study is important as a first step in screening the anticancer activity of methanol extract of* A. chinensis. *We further intend to isolate these active ingredients and decipher their anticancer mechanisms.

In conclusion, the methanolic extract of* A. chinensis* can inhibit the proliferation of MDA-MB-453 and HCC-1937 cells by inducing cell cycle arrest; the oleic acid and palmitic acid were found to be the main compounds in AME. These results suggest that AME can be a potential natural alternative or can provide a complementary therapy for breast cancer. Further evaluation of the anticancer properties of AME* in vivo* is needed to ensure its efficacy and long-term safety.

## Figures and Tables

**Table 1:** Primer sequences used for real-time RCR analysis.

Gene	Forward primer	Reverse primer
GAPDH	5′-CCACTCCTCCACCTTTG-3′	5′-CACCACCCTGTTGCTGT-3′
GADD45A	5′-CGAGAACGACATCAACA-3′	5′-CGGCAAAAACAAATAAG-3′
CDC20	5′-TCTGGTCTCCCCATTACA-3′	5′-ATAGCCTCAGGGTCTCAT-3′
AURKB	5′-AGAGATGATTGAGGGGCG-3′	5′-GATTGAAGGGCAGAGGGA-3′
PLK1	5′-TAAGTCTCTGCTGCTC-3′	5′-ATAACTCGGTTTCGGT-3′
CCNB2	5′-CAACCCACCAAAACAACA-3′	5′-GCAAGGCATCAGAAAAAG-3′
TOP2A	5′-ATAGGAGCAGTGACGA-3′	5′-GGAAATGTGTAGCAGG-3′

**Table 2:** Compounds detected by GC-MS analysis of methanol extracts from *Aspongopus chinensis* Dallas.

Sample NO.	RT (min)	Name of compounds	MF	CS	MW	Area (%)
1	38.678	oleic acid	C_18_H_34_O_2_	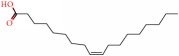	282.461	25.39
2	35.348	palmitic acid	C_16_H_32_O_2_	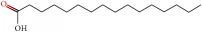	256.424	21.798
3	34.924	z-11-Hexadecenoic acid	C_16_H_30_O_2_	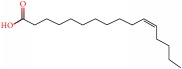	254.408	8.077
4	37.327	threitol, acetylated	C_12_H_18_O_8_	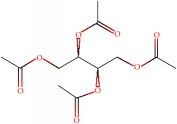	290.267	6.739
5	39.008	Stearic acid	C_18_H_36_O_2_	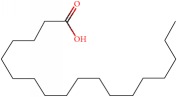	284.477	3.036
6	11.745	2-Hexenoic acid	C_6_H_10_O_2_	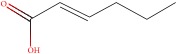	114.142	2.491
7	45.873	1-(14-methylhexadecanoyl) pyrrolidine	C_21_H_41_NO	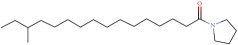	323.556	0.344

RT, retention time; MF, molecular formula; CS, chemical structure; MW, molecular weight. The chemical structure was made using the National Institute of Standard and Technology (NIST) Library.

## Data Availability

The data used to support the findings of this study are available from the corresponding author upon request.
